# Ischemic colitis induced by indigo naturalis in a patient with ulcerative colitis: a case report

**DOI:** 10.1186/s12876-020-01301-3

**Published:** 2020-05-15

**Authors:** Byungha Cho, Soon Man Yoon, Seung-Myoung Son, Hyoung Woo Kim, Ki Bae Kim, Sei Jin Youn

**Affiliations:** 1 Department of Internal Medicine, Chungbuk National University Hospital, Chungbuk National University College of Medicine, 776, 1 Sunhwan-ro, Seowon-gu, Cheongju, 28644 Korea; 2 Department of Pathology, Chungbuk National University Hospital, Chungbuk National University College of Medicine, Cheongju, Korea

**Keywords:** Ulcerative colitis, Ischemic colitis, Indigo naturalis, Adverse event, Case report

## Abstract

**Background:**

Indigo naturalis is a Chinese herbal medicine that has currently been used to treat various inflammatory diseases, including ulcerative colitis. Recently, there are several reports concerning severe adverse events associated with indigo naturalis.

**Case presentation:**

We described a case of a 44-year-old female with ulcerative colitis who presented with lower abdominal pain and hematochezia. She stopped taking her medicine for ulcerative colitis and started oral indigo naturalis 3 months before admission. Computed tomography showed segmental edematous wall thickening of the descending and sigmoid colon. Colonoscopy findings revealed erythema, edema, and submucosal hemorrhage, the surface of which presented a dark blue pigmentation. The histologic finding was consistent with ischemic colitis. We therefore considered an ischemic colitis induced by indigo naturalis, and the patient improved after supportive care and withdrawal of indigo naturalis.

**Conclusion:**

Indigo naturalis has currently been used in the patients with ulcerative colitis as an alternative therapy. However, physicians should be aware of possible severe adverse events such as ischemic colitis.

## Background

Indigo naturalis (also known as Qing-dai) is a Chinese herbal medicine with a dark blue color, which is extracted from the leaves and stems of plants such as *Indigofera tinctoria*, *Strobilanthes cusia O Kuntze*, and *Polygonum tinctorium Lour*. Indigo naturalis has been used in China for centuries to treat various inflammatory diseases [1, 2].

There have recently been several reports suggesting that indigo naturalis is effective in patients with active ulcerative colitis, although the evidence regarding efficacy and safety is still limited [3–6]. Common adverse effects of indigo naturalis are liver dysfunction, headache, abdominal pain, and nausea [3–6]. Recently, there have been concerns about severe adverse events like pulmonary arterial hypertension and acute colitis in patients taking indigo naturalis [7–12].

We herein report a patient with ulcerative colitis who developed ischemic colitis during oral administration of indigo naturalis.

## Case presentation

A 44-year-old woman with a two-year history of ulcerative colitis was admitted to our hospital with complaints of lower abdominal pain and hematochezia. The patient had previously been treated with oral mesalazine and intermittent corticosteroids at another hospital, and the disease extent was the extensive colitis involved to the transverse colon. The patient had no other medical history, including hypertension, diabetes, cardiovascular disease, chronic kidney disease or abdominal surgery.

**Fig. 1:**
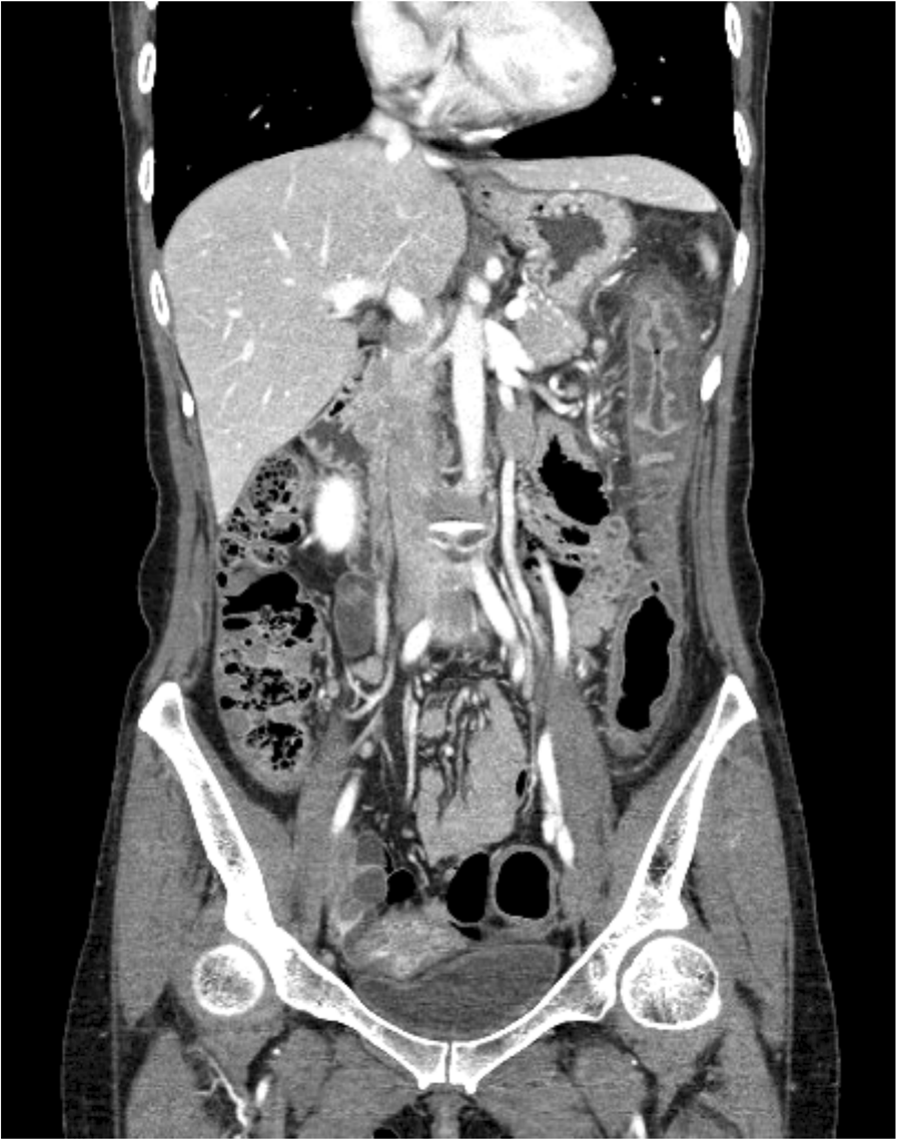
Computed tomography demonstrating segmental edematous wall thickening of the descending and sigmoid colon

**Fig. 2:**
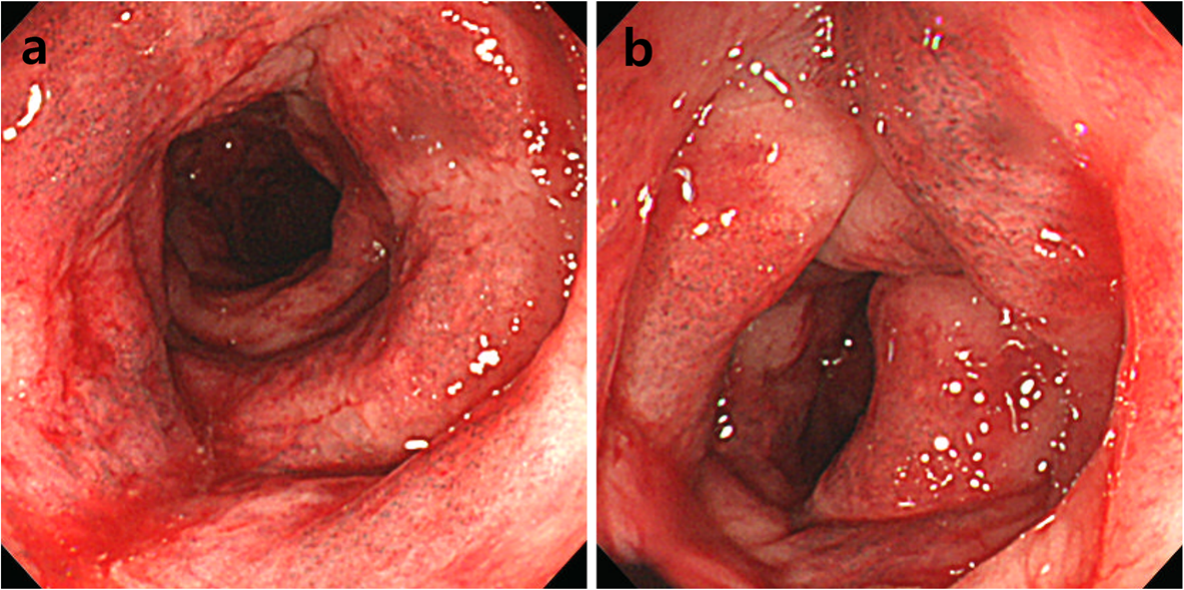
Colonoscopy showing erythema, edema, and submucosal hemorrhage, the surface of which presents a dark blue pigmentation with the same color as the original indigo naturalis in the descending (**a**) and sigmoid colon (**b**)

**Fig. 3:**
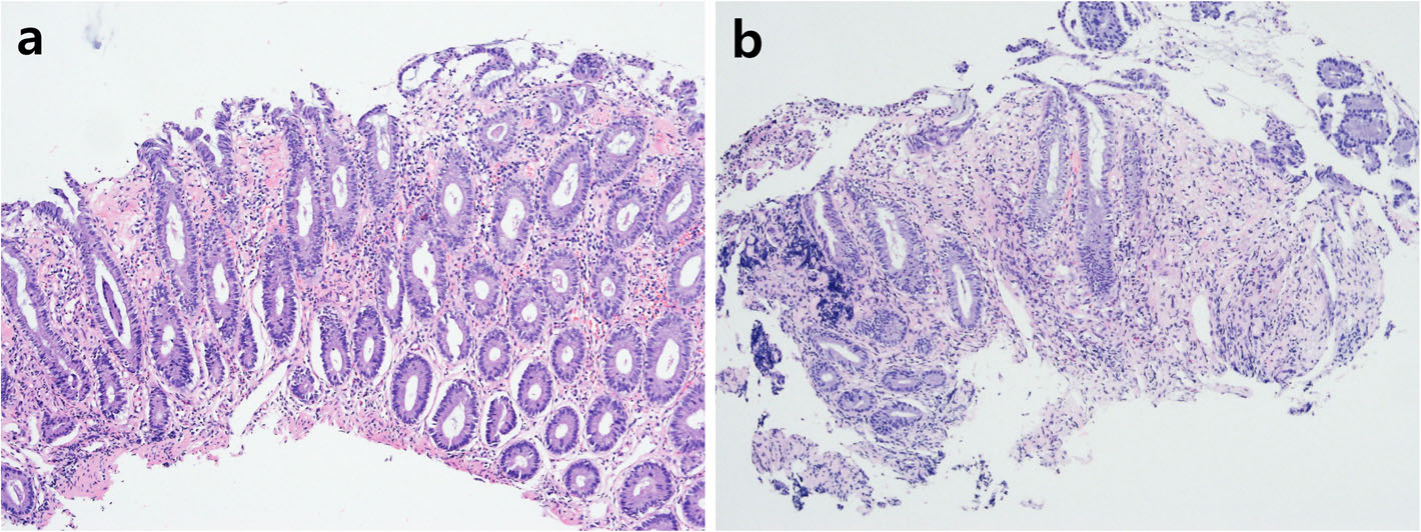
Pathologic findings of colonoscopic biopsies demonstrating erosion and loss of superficial epithelium with few inflammatory infiltrations (hematoxylin-eosin staining, × 100)

**Fig. 4:**
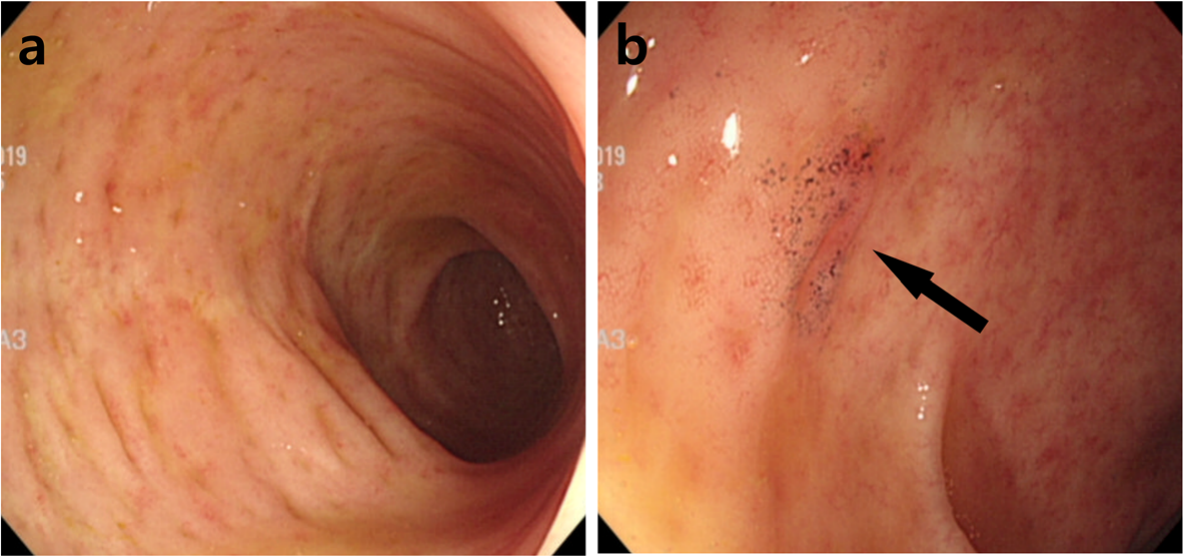
Follow-up colonoscopy showing an improved state with some scar changes and indigo blue pigmentation (arrow) still remaining on the descending (**a**) and sigmoid colon (**b**)

Three months prior to admission, she independently stopped taking her medication for ulcerative colitis and started taking self-purchased oral indigo naturalis twice a day (daily dose, 2 g). She had no history of taking other medication. After 2 months of taking the oral indigo naturalis, she developed intermittent hematochezia. Two weeks later, she exhibited lower abdominal pain. The patient’s vital signs were stable on admission. Her physical examination revealed tenderness on lower abdomen, but not rebound tenderness. Laboratory examination on admission showed a white blood cell count of 9.4 × 10^3^/μL (reference, 4000-10,000/μL), hemoglobin level of 13.2 g/dL (reference, 12–16 g/dL for female), and C-reactive protein concentration of 0.45 mg/dL (reference, < 0.3 mg/dL). A stool culture and *Clostridium difficile* toxin study were both negative. Computed tomography of abdomen and pelvis showed segmental edematous wall thickening of the descending and sigmoid colon, and no abnormal finding of mesenteric vessels (Fig. 1). Colonoscopy findings revealed erythema, edema, and submucosal hemorrhage, the surface of which presented a dark blue pigmentation with the same color as the original indigo naturalis in the descending and sigmoid colon, but a relatively non-inflamed finding in the rectum (Fig. 2a, b). Biopsy specimens from the descending and sigmoid colon showed erosion and loss of superficial epithelium with few inflammatory infiltrations, which is histologically consistent with ischemic colitis rather than a flare-up of ulcerative colitis or other colitis such as infectious colitis (Fig. 3a, b). Based on these findings, we considered the patient to have ischemic colitis induced by indigo naturalis. Supportive care with bowel rest, intravenous hydration, empirical antibiotics, and discontinuation of indigo naturalis resulted in improvement within a week. She then agreed to be treated with prednisolone and mesalazine for ulcerative colitis, and a follow-up colonoscopy 2 months later showed an improved state with some scar changes and an indigo blue pigmentation still remaining on the colon (Fig. 4a, b).

## Discussion and conclusion

Indigo naturalis has been considered mainly as an anti-fever and anti-inflammatory agent in Chinese textbooks since the tenth century, and has therefore been used to treat various inflammatory diseases for centuries [1, 2]. There have recently been studies suggesting the efficacy of indigo naturalis in patients with active ulcerative colitis [3–6]. The actual mechanisms of action of indigo naturalis still remain unknown, but one possible mechanism is that the active components of indigo naturalis can activate the aryl hydrocarbon receptor (AhR) by acting as an AhR ligand, and promote mucosal healing by the upregulation of interleukin-22 from innate lymphocytes cells that express AhR [2, 13].

The first study to evaluate the therapeutic efficacy of oral indigo naturalis in patients with intractable ulcerative colitis showed that clinical activity index scores and endoscopic grades decreased in the patients taking 2 g of indigo naturalis daily for 4 months [3]. In 2018, Naganuma et al. conducted a randomized controlled trial in Japan to investigate the clinical efficacy and safety of indigo naturalis in patients with moderate to severe ulcerative colitis [5]. Total of 86 patients were enrolled and randomized for each group, the results indicated that 8 weeks of oral indigo naturalis produced a significant clinical response in the indigo naturalis groups. The response rates at week 8 were 69.6% (16/23, 0.5 g/day group), 75.0% (15/20, 1.0 g/day group), 81.0% (17/21, 2.0 g/day group), and 13.6% (3/22, placebo group), respectively. However, this trial was terminated early because a case of pulmonary arterial hypertension was reported in a patient with ulcerative colitis who had used self-purchased indigo naturalis outside of this study [5, 7].

Some patients have recently started using indigo naturalis obtained over the Internet, but these patients do not always receive sufficient information regarding the adverse effects caused by indigo naturalis. Common mild adverse events of indigo naturalis include liver dysfunction, headache, epigastric or abdominal pain, and nausea [3–6]. Rare severe adverse events associated with indigo naturalis have included indigo naturalis-induced pulmonary arterial hypertension and acute colitis [7–12]. Nishio et al. reported the development of pulmonary arterial hypertension in a patient with ulcerative colitis who was taking indigo naturalis [7]. An experimental study suggested that a possible mechanism for triggering indigo naturalis-induced pulmonary arterial hypertension involves nitric oxide synthase inhibition and endothelial dysfunction in the pulmonary artery [8]. Recently, indigo naturalis-induced colitis has also been reported. A prospective study about clinical efficacy and safety of indigo naturalis in patients with ulcerative colitis described right-side colitis of obscure origin [4]. Kondo et al. also reported two patients with ulcerative colitis who developed colitis with wall thickening and edematous changes during oral administration of indigo naturalis [10].. In addition, Matsuno et al. reported two patients who developed phlebitis-induced colitis caused by indigo naturalis. They suggested that indigo naturalis might affect the venous system and cause adverse events such as phlebitis-induced colitis or pulmonary artery hypertension [12].

We herein described a case of ischemic colitis in a patient with ulcerative colitis after taking indigo naturalis. About 30 to 50% of patients with inflammatory bowel disease are known to use complementary and alternative medicines [14]. Thus, detailed medical history is needed when treating patients with inflammatory bowel disease. Indigo naturalis seems to be another therapeutic candidate for managing ulcerative colitis, but more research is needed to determine the optimal dose, standardized drug development, and indications for treatment. In addition, physicians should be aware of the possible risks of severe adverse events. Further basic studies and clinical investigation are needed to clarify the mechanisms of these possible adverse events of indigo naturalis.

## Data Availability

This case report contains clinical data from the electronic medical record in the Chungbuk National University Hospital. Additional information is available from the corresponding author on reasonable request from the editor.

## Abbreviation

AhR: Aryl hydrocarbon receptor
